# Wheat leaf area index prediction using data fusion based on high-resolution unmanned aerial vehicle imagery

**DOI:** 10.1186/s13007-022-00899-7

**Published:** 2022-05-19

**Authors:** Shuang Wu, Lei Deng, Lijie Guo, Yanjie Wu

**Affiliations:** 1 College of Resource Environment and Tourism, Capital Normal University, Beijing, 100048 China; 2 Engineering Research Center of Spatial Information Technology, Ministry of Education, Capital Normal University, Beijing, 100048 China; 3 Beijing Laboratory of Water Resources Security, Capital Normal University, Beijing, 100048 China

**Keywords:** Leaf area index (LAI), Unmanned aerial vehicle (UAV), High resolution, Data fusion

## Abstract

**Background:**

Leaf Area Index (LAI) is half of the amount of leaf area per unit horizontal ground surface area. Consequently, accurate vegetation extraction in remote sensing imagery is critical for LAI estimation. However, most studies do not fully exploit the advantages of Unmanned Aerial Vehicle (UAV) imagery with high spatial resolution, such as not removing the background (soil and shadow, etc.). Furthermore, the advancement of multi-sensor synchronous observation and integration technology allows for the simultaneous collection of canopy spectral, structural, and thermal data, making it possible for data fusion.

**Methods:**

To investigate the potential of high-resolution UAV imagery combined with multi-sensor data fusion in LAI estimation. High-resolution UAV imagery was obtained with a multi-sensor integrated MicaSense Altum camera to extract the wheat canopy's spectral, structural, and thermal features. After removing the soil background, all features were fused, and LAI was estimated using Random Forest and Support Vector Machine Regression.

**Results:**

The results show that: (1) the soil background reduced the accuracy of the LAI prediction of wheat, and soil background could be effectively removed by taking advantage of high-resolution UAV imagery. After removing the soil background, the LAI prediction accuracy improved significantly, R^2^ raised by about 0.27, and RMSE fell by about 0.476. (2) The fusion of multi-sensor synchronous observation data could achieve better accuracy (R^2^ = 0.815 and RMSE = 1.023), compared with using only one data; (3) A simple LAI prediction method could be found, that is, after selecting a few features by machine learning, high prediction accuracy can be obtained only by simple multiple linear regression (R^2^ = 0.679 and RMSE = 1.231), providing inspiration for rapid and efficient LAI prediction of wheat.

**Conclusions:**

The method of this study can be transferred to other sites with more extensive areas or similar agriculture structures, which will facilitate agricultural production and management.

## Background

Wheat is the most widely grown grain crop in the world, and it plays an essential role in the food supply, accounting for approximately 20% of total energy consumption [1–3]. As the primary photosynthetic organ, the leaves of wheat have a significant impact on the overall growth. Leaf area index (LAI), as an essential parameter of wheat growth, can provide dynamic information during wheat growth. It is a critical metric for assessing crop growth and is closely related to the aboveground biomass and yield [4–7]. As a result, rapid, accurate, and non-destructive prediction of LAI is critical for field management. The traditional method of obtaining LAI is through artificial ground destructive sampling, which is time-consuming, labor-intensive, and hinders crop growth. The advancement of remote sensing technology in recent years has provided a new means for the rapid acquisition of LAI [8, 9].

Because LAI is half the amount of leaf area per unit horizontal ground surface area [10], accurate vegetation extraction in remote sensing imagery is critical. The vegetation information of crop canopy extracted from imagery is primarily determined by the combined effects of vegetation types, soil properties, shadows, and other factors [11]. The background (soil, weeds, and shadow) accounts for a certain proportion of the plot area in crop growth. The soil background exists throughout the crop growth cycle. Not only does the soil background account for a large proportion of the plot area in the early stages of crop growth, but exposed soil is also found in the late stages of crop growth due to differences in some factors (such as seedling emergence rate) between crops. Previously, some researchers attempted to estimate crop LAI using satellite remote sensing imagery, such as Landsat and Sentinel-2 satellite imagery [12–14]. Kamenova et al. [13] used various vegetation indices (VIs) extracted from Sentinel-2 multispectral imagery to establish the LAI prediction model, and LAI of winter wheat can be better estimated. Meyer et al. [14] found that the raw value of bands and VIs extracted from Landsat8-OLI multispectral imagery were used to establish a prediction model that can effectively predict the LAI of the temperate deciduous broad-leaved forest.

It can be seen that research on satellite remote sensing has made progress. However, satellite imagery was limited by coarse spatial resolution. The extracted vegetation information is usually mixed with background information such as soil, resulting in the wrong calculation of leaf area per unit horizontal surface area. Thus, the estimated LAI value of the crop is inaccurate. In recent years, Unmanned Aerial Vehicle (UAV) remote sensing can obtain centimeter-level high spatial resolution imagery, which is useful for distinguishing vegetation, and background information, and is often used to estimate crop traits [15–17]. However, most UAV remote sensing studies currently still use the way of satellite remote sensing, which means that the extracted vegetation information without background processing is directly used in crop trait estimation, resulting in inaccurate LAI estimation [18, 19]. Fortunately, some studies have noted the influence of the soil background. The remote sensing imagery of the UAV is processed in advance to remove the soil background. Still, it is only applied to estimating the crop's chlorophyll content [16] or yield [20], and the estimation of the crop's LAI is little. Furthermore, previous research has shown that it is difficult to observe a variety of data synchronously using UAV remote sensing. To address this issue, the typical solution is to carry multiple sensors (multispectral and thermal infrared sensors, etc.) on the same UAV platform and calibrate the generated multiple imageries using ground control points (GCPs), which is inefficient. For example, Maimaitijiang et al. [20], the Mapir Survey2 RGB and FLIR Vue Pro r 640 cameras were installed on the DJI S100 + UAV platform to obtain visible and thermal infrared imagery, respectively. And then, GCPs were used to calibrate two kinds of images to obtain spectral and thermal information. The integration of tiny sensors on UAV is increasing rapidly, UAV can be equipped with multiple imaging sensors and GPS systems to obtain many data sets (RGB imagery, 3D points clouds and thermal imagery, etc.) simultaneously. It has become one of the most competitive tools, providing excellent possibilities for precision agriculture [21–23]. A typical example, such as the Micasense Altum camera, is the integration of multispectral and thermal infrared cameras into a single unit, which has the advantage of overlapping fields of view and simultaneous access to canopy spectral, structural, and thermal information [24]. Although a variety of data can be obtained by using the sensors integrated with UAV, few studies on their comprehensive utilization/fusion and potential in LAI application is still unknown.

Many regression methods based on statistics and machine learning, such as Partial Least Squares Regression (PLSR) [25], Artificial Neural Network (ANN) [26], and Random Forest Regression (RFR) [27], are currently used in crop trait estimation to realize the fusion of multi-sensor data [18]. However, most studies tend to add all the features extracted from remote sensing data to the model for training in data fusion using machine learning methods. The advantages of this are that it considers all the features, but it does not consider how adding them may affect the model's efficiency and lengthen its running time [28, 29]. Previous studies have found different important features for different crops [18, 20]. For example, Lee et al. [18] found that when using RFR to predict the nitrogen content of maize, the VIs such as Modified Simple Ratio (MSR), Wide Dynamic Range Vegetation Index (WDRI), and Ratio Vegetation Index (RVI) performed well. Maimaitijiang et al. [29] discovered that canopy height and vegetation coverage extracted from UAV imagery performed well in estimating aboveground biomass and LAI of soybean. However, there have been few studies on the critical features of wheat LAI estimation [30]. Furthermore, most studies have shown that Multiple Linear Regression (MLR) is used to estimate crop traits rapidly. Because it has fast modeling speed, does not require very complex calculations, and still runs fast in the case of large amounts of data. However, there are few studies on the potential of MLR in the rapid estimation of wheat LAI, and few can propose a simple model for wheat LAI, that is, using a few important features with MLR.

The main purpose of this study is to find a method to estimate LAI with high precision by making full use of the advantages of UAV remote sensing, which means using the advantage of high spatial resolution of UAV imagery combined with multi-sensor synchronous observation. The specific objectives are as follows: (1) to investigate the influence of soil background on LAI estimation using the high-resolution UAV imagery; (2) to evaluate the potential of data fusion based on multi-sensor in LAI estimation and find an efficient and straightforward LAI prediction method.

## Materials

### Test site and filed layout

**Fig. 1:**
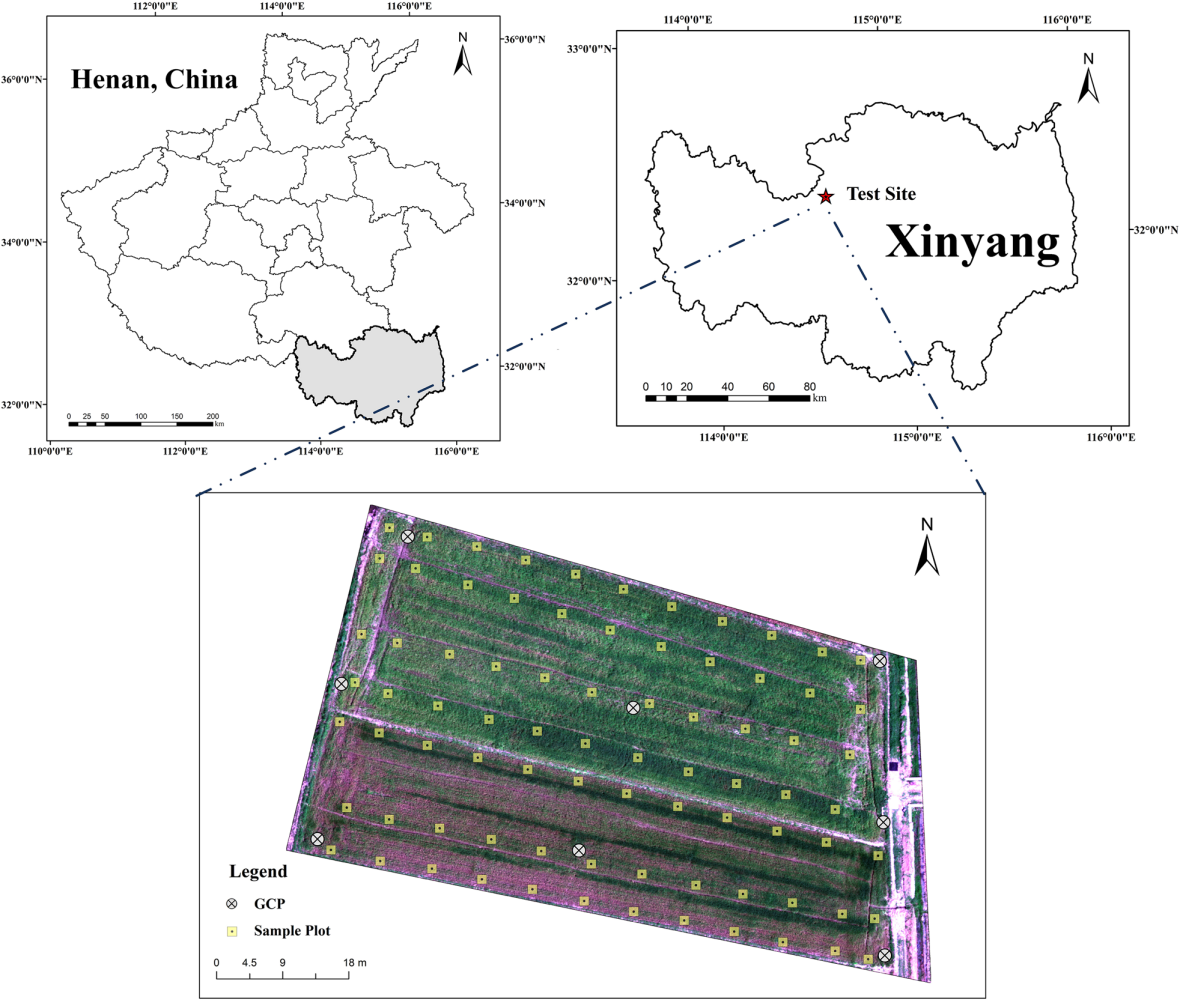
Test site of the wheat fields shown using red, green, blue (RGB) sensor mosaic imagery taken on 1 May in Xinyang County, Henan Province, China

The study area is located in wheat-growing farmland in Xi county, Henan Province, China, and covers an area of approximately 3565.497 m^2^, as shown in Fig. 1. The climate in the region is subtropical monsoon, with an annual average temperature of 15.5 ℃, average yearly rainfall of more than 1200 mm, and a daily average temperature of 10 ℃. As a result, it is ideal for wheat planting and growth in this case. The wheat was planted in October and harvested the following June. The experiment was conducted here on May 1, 2021. Currently, wheat was in the heading stage of the growth period, with dark green leaves. To further facilitate establishing and verifying the LAI prediction model, 80 2 m × 2 m plots were designed, and the ground data was measured on 80 plots. At the same time, 8 GCPs are arranged in the study area for UAV imagery correction and registration, as shown in Fig. 1.

### Data and processing

#### Field data acquisition

**Fig. 2:**
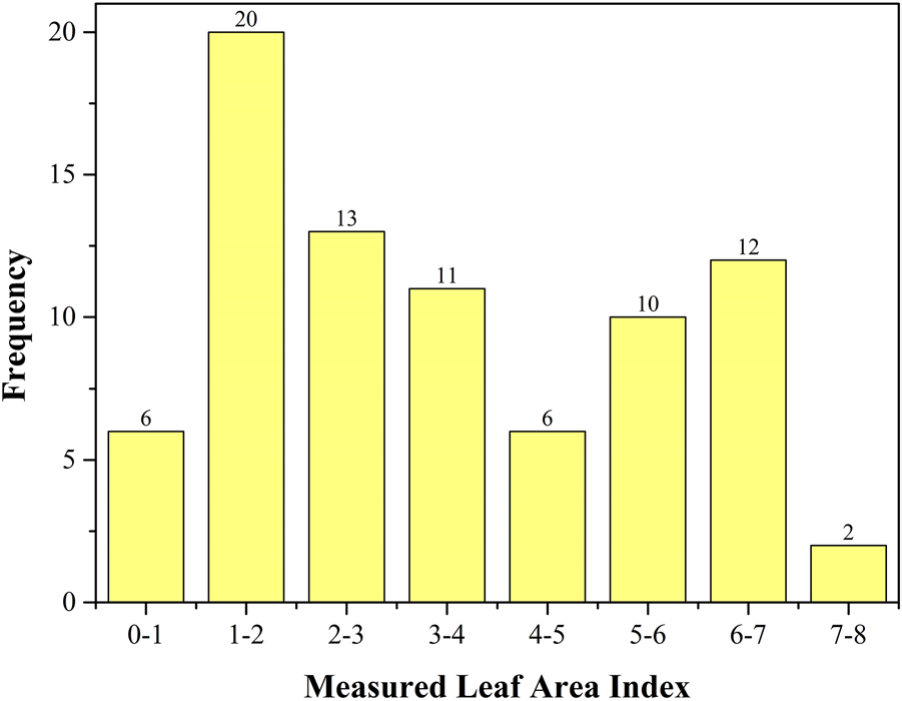
Number of plots with different measured leaf area index area

LAI of 80 plots was measured by LAI-2200C Plant Crown Analyzer (LI-COR Inc., Lincoln, NE, USA) on May 1, 2021. The field data are obtained according to the measurement guidelines recommended in the instrument manual. 23 LAI values are randomly collected from each plot and then averaged to represent the LAI values of each plot. The number of plots within a certain LAI value range is counted, as shown in Fig. 2. It is shown that the LAI of 80 plots is different. The LAI values are concentrated in the ranges of 1–2, 2–3, and 6–7, with 20, 13, and 12, respectively. The LAI value is in the range of 7–8, and the number of plots is two.

#### UAV imagery acquisition and processing

The UAV imagery was obtained at 10:24 a.m. on May 1, 2021, when the weather was clear and cloudless. This study used MicaSense Altum (Seattle, WA, USA) sensor installed on DJI Matrice200 four-axis aircraft. The MicaSense Altum has five high-resolution multispectral bands (blue, green, red, red edge, and near-infrared) and integrated long-wave thermal infrared (TIR) sensor (based on the FLIR Lepton), which is aligned with the multispectral sensors. The specific spectral parameters of the camera are shown in Table 1. The TIR sensor recalibrates every 5 min or when a 2 K change in temperature occurs. The reported accuracy is ± 5 K with thermal sensitivity of < 50 mK. The flight altitude of the UAV is set at 30 m, the heading overlap is 80%, and the side overlap is 70%.

**Table 1:** Spectral characteristics of the six MicaSense bands

Band#	Name	Center wavelength(nm)	Bandwidth(nm)
1	Blue	475	32
2	Green	560	27
3	Red	668	14
4	Red edge	717	12
5	Near-infrared (NIR)	842	57
6	Thermal infrared	Band Range (μm): 8–14	

The UAV images were processed using a photogrammetry software called Pix4D mapper (Pix4D SA, Lausanne, Switzerland). Pix4Dmapper was used to generate an orthomosaic image of each field by stitching hundreds of different images captured during the same flight into one single 2D image and correcting for perspective. The mosaic images were automatically radiometrically corrected in Pix4D with a spatial resolution of 1.49 cm/pixel. Pix4D uses the Structure from Motion (SfM) technique and has been well-suited for UAV data as it combines images from multiple angles. In addition, the geographic coordinates of the eight ground control points were used during the photogrammetric workflow of Pix4Dmapper to improve the vertical and horizontal accuracy of the output orthomosaics.

## Methods

**Fig. 3:**
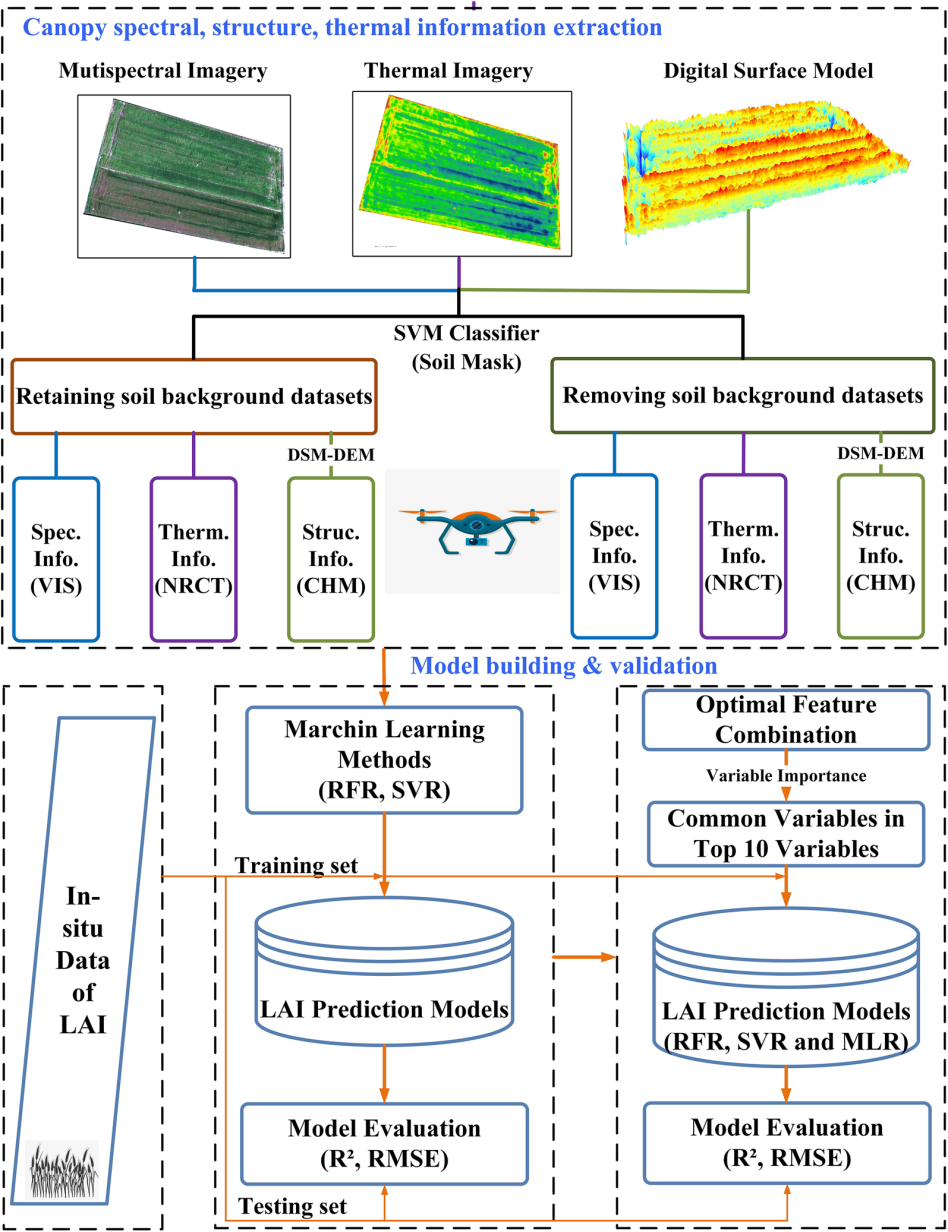
A workflow diagram of data processing, feature extraction and LAI prediction model building and validation

The workflow (Fig. 3) that we used in this study was divided into two sections. The first section was feature extraction, preparing the input variables for the LAI prediction model; the second section was LAI prediction model building and validation, which could find important variables and a fast LAI prediction method.

Firstly, to investigate the influence of soil background and the potential of data fusion of multi-sensor synchronous observation in LAI prediction. The UAV high spatial resolution dataset was divided into two parts: one with the soil background removed and one with the soil background retained. The canopy's spectral, structural, and thermal features were extracted from the dataset. Second, after fusing the spectral, structural, and thermal features, machine learning methods (RFR and SVR) were used to model. The model's accuracy was then evaluated to find the optimal feature combination. Finally, to find a rapid and efficient LAI prediction method, each variable's importance in the model was ranked based on the optimal feature combination. The common variables among the top ten variables were selected. MLR was used to model, and the model's accuracy was evaluated.

### Feature extraction

**Fig. 4:**
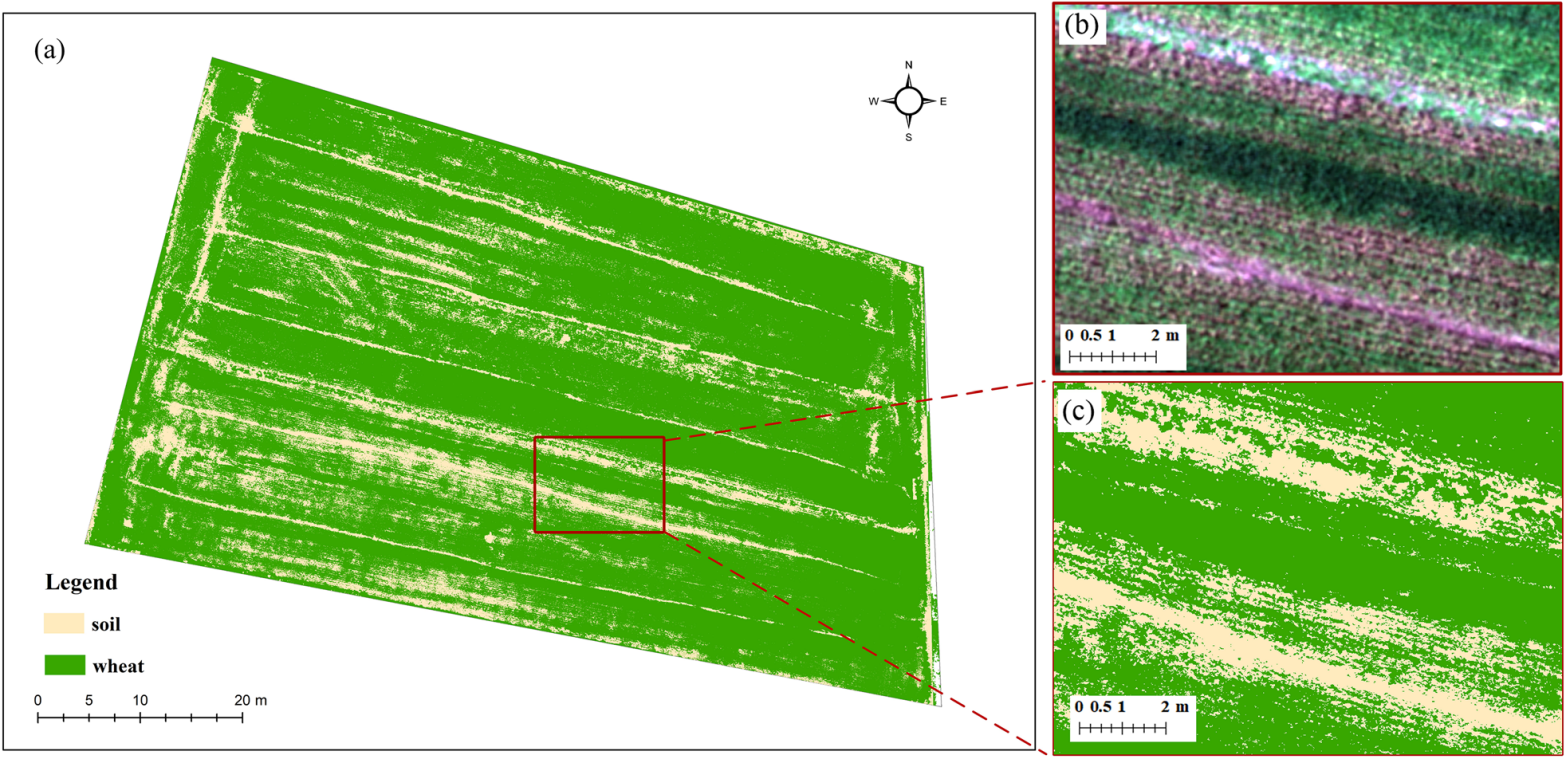
Vegetation fraction and soil removal. **a** shows the entire field, **b** is a close-up RGB image, and **c** shows the corresponding vegetation and soil map of the close-up view

To explore the potential of multi-sensor synchronous observation data in LAI prediction, it is mainly divided into three parts: canopy spectral, structure, and thermal information. The data set needs to be processed simply before feature extraction, and the specific operation is as follows: firstly, two datasets were prepared. For one dataset, the soil background was retained, which means no background processing is done on dataset, referred to as the dataset with soil background; for another dataset, the soil background was removed, referred to as the dataset without soil background. The process of removing the soil background is as follows: display the UAV multispectral imagery in the true color composite of the blue, green, and red bands. Support Vector Machine (SVM) classifier was used to identify wheat and soil on the UAV imagery. A binary mask layer was established to exclude background soil pixels from all spectral, structure, thermal, and texture features extracted from UAV imagery for further processing. The performance of the SVM classifier was evaluated using the confusion matrix and accuracy statistics overall accuracy (OA) and Kappa coefficient based on randomly selected independent test samples. The SVM classification resulted in an OA of 98.1% and a Kappa coefficient of 0.976 (Fig. 4).

For the two datasets with and without soil background, the following same processing is performed. Average pixel values for each of the spectral, structure, and thermal raster layers listed in Table 2 were computed at a small yield plot level using zonal statistics to relate them with the corresponding wheat LAI. The Arcpy library and Python 2.7 programming language were used to apply zonal statistics, remove soil background, and automate and streamline the extraction of raster layers, the extracted features as shown in Table 2.

**Table 2:** Definitions of the features extracted from different imagery

Feature	Features	Formulation	References
Spec. Info	Blue (B), Green (G), Red (R), Red-Edge (RE), Near-infrared (NIR)	The raw value of each band	–
	Ratio vegetation index	RVI = NIR/R	[31]
	Green chlorophyll index	GCI = (NIR/G)−1	[32]
	Red-edge chlorophyll index	RECI = (NIR/RE)−1	[32]
	Normalized difference vegetation index	NDVI = (NIR−R)/(NIR + R)	[33]
	Green normalized difference vegetation index	GNDVI = (NIR−G)/(NIR + G)	[34]
	Green–red vegetation index	GRVI = (G−R)/(G + R)	[31]
	Normalized difference red-edge	NDRE = (NIR−RE)/(NIR + RE)	[35]
	Normalized difference red-edge index	NDREI = (RE−G)/(RE + G)	[36]
	Simplified canopy chlorophyll content index	SCCCI = NDRE/NDVI	[37]
	Optimized soil adjusted vegetation index	OSAVI = (NIR−R)/(NIR + R + L) (L = 0.16)	[38]
	Modified chlorophyll absorption in reflectance index	MCARI = [(RE−R)−0.2*(RE−G)] *(RE/R)	[39]
	Transformed chlorophyll absorption in reflectance index	TCARI = 3*[(RE−R)−0.2*(RE−G) *(RE/R)]	[40]
	MCARI/OSAVI	MCARI/OSAVI	[39]
	TCARI/OSAVI	TCARI/OSAVI	[40]
	Wide dynamic range vegetation index	WDRVI = (a*NIR−R)/(a*NIR + R) (a = 0.12)	[41]
Struc. Info	Canopy Height Model (m)	CHM = DSM−DEM	/
Therm. Info	Normalized relative canopy temperature index	$NRCT = \frac{{T_{i} - T_{\min } }}{{T_{\max } - T_{\min } }}$	[42]

#### Canopy spectral information

The original bands (Blue, Green, Red, Red Edge, and Near infrared) from multispectral orthomosaics were used as canopy spectral features. In addition, a group of VIs was used for crop monitoring and trait estimation as usual, such as NDVI, RVI, and NDRE, were selected (Table 2).

#### Canopy structure information

The canopy height model (CHM) was extracted from photogrammetric point clouds and used as canopy structure features to predict LAI in this study. To obtain CHM, the digital surface model (DSM) and the digital elevation model (DEM) [43, 44], which were created by photogrammetric 3D point clouds, were obtained firstly. Because DSM represents the height of the ground and all objects on the ground, while DEM only represents the height of the ground, the CHM could be obtained by subtracting DEM from DSM. An essential step in creating DEM and DSM is to identify Ground Points (GP) and Non-Ground Points (NGP) in dense point clouds, which is performed in Pix4Dmapper software. This tool allows distinguishing between GP, NGP, and noise points. DSM is created from all categories except noise points, while DTM is calculated only from points identified as GP.

#### Canopy thermal information

**Fig. 5:**
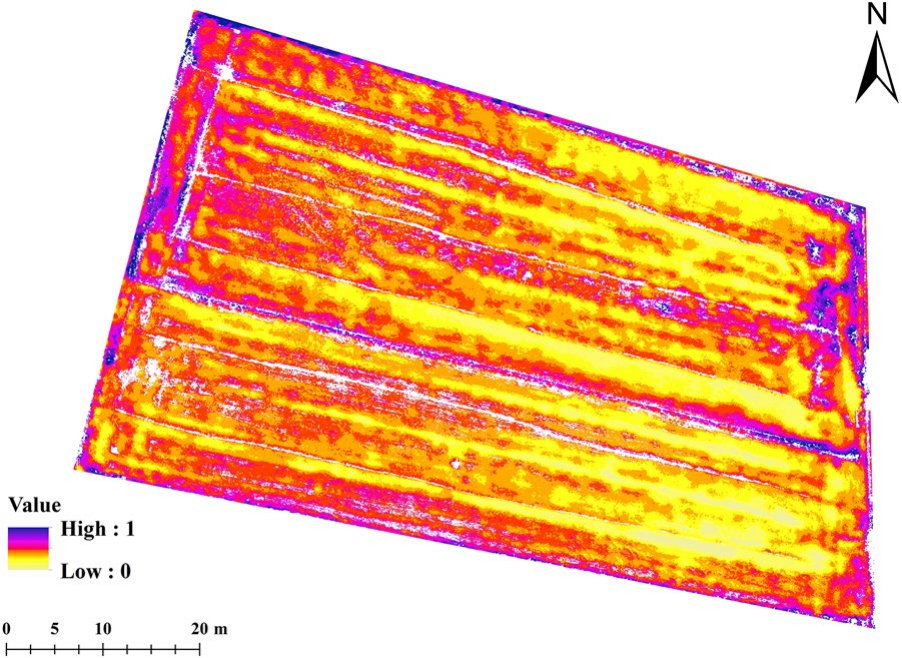
Normalized relative canopy temperature index distribution map (removed soil pixels are represented by white color)

To facilitate the use of temperature data, it is normalized and mapped to the range of 0–1. The thermal feature of LAI prediction, the Normalized Relative Canopy Temperature (NRCT) [42], was calculated from thermal infrared images. NRCT was computed using the canopy temperature in the imagery, the minimum temperature (T_min_, lower baseline), and the maximum temperature (T_max_, upper baseline) of the whole study area, as shown in Fig. 5. NRCT has been used to evaluate water status and crop traits. The larger the value of NRCT, the higher the temperature; the smaller the value of NRCT, the lower the temperature [42, 45]. The NRCT was calculated based on the following equation:

1 $$NRCT = \frac{{T_{i} - T_{\min } }}{{T_{\max } - T_{\min } }}$$

where *T*_*i*_ represents the canopy temperature of the *i* th pixel, *T*_*min*_ is the lowest temperature in the whole field trial, and *T*_*max*_ the highest temperature in the whole field trial.

### LAI prediction model building and validation

Several machine learning methods were used in remote sensing applications, especially in crop monitoring and trait estimation, such as RFR and SVR. This study used RFR and SVR to estimate the LAI based on canopy spectrum, structure, and thermal features. RFR is a nonparametric integration method based on the Classification and Regression Tree (CART). It is made up of different trees that have been trained using bagging and random variable selection, which is more tolerant of outliers and noise [46, 47]. The number of decision trees determines the performance of RFR. The number of decision trees tested was 50, 100, 150, and 200. To strike a balance between calculation time and accuracy, the number of decision trees was finally set at 100. SVR is a form of nonparametric modeling that defines boundaries in a high-dimensional space using a hyperplane [48, 49]. To construct an SVR with good performance, the selection of kernel function is the key, so the linear kernel function is selected [48].

First, multiple features are fused, and then the fused features are used as input features of the model. To better train and evaluate the model, 70% randomly selected input features and LAI were used as training samples, and the remaining 30% were used as unseen samples to test the performance of the model. The coefficients of determination (R^2^) and root mean square error (RMSE) were computed to evaluate the performance of the LAI prediction model and can be expressed as follows:

2 $$R^{2} = 1 - \frac{{\sum\nolimits_{i = 1}^{n} {(y_{i} - \widehat{{y_{i} }})^{2} } }}{{\sum\nolimits_{i = 1}^{n} {(y_{i} - \overline{y} )^{2} } }}$$

3 $$RMSE = \sqrt {\frac{{\sum\nolimits_{i = 1}^{n} {(y_{i} - \widehat{{y_{i} }})^{2} } }}{n}}$$

where *y*_*i*_ and $\widehat{{y_{i} }}$ are the measured and the predicted LAI, respectively. $\overline{y}$ is the mean of measured LAI, and *n* is the total number of samples in the testing set.

To estimate LAI quickly and efficiently, the best feature combination was determined by comparing the model's accuracy. For the best feature combination, according to the importance of variables in the RFR and SVR models, common variables were found in the top ten variables of the two models. And then, MLR was to build the model based on common variables. It is known that the MLR is to predict or estimate the dependent variable by the optimal combination of multiple independent variables, which is more effective and practical than using only one independent variable [50]. The IBM SPSS Modeler 18.0 was used to build the LAI prediction model and variable importance ranking.

MLR is one of the statistical methods, which attempts to model the correlation between involving variables and a response variable depending on linear equation into the observed data. Compared with most regression methods of machine learning, it is simpler and easier to operate. The MLR model is:

4 $$y_{i} = b_{0} + b_{1} x_{i,1} + b_{2} x_{i,2} + \cdots + b_{k} x_{i,k} + e_{i}$$

where, *y*_*i*_ is the dependent variable; *b*_*0*_ is the intercept; *x*_*i,k*_ is an independent variables; *b*_*k*_ is the vector of regression coefficients; and *e*_*i*_ is random measured errors.

## Results

### Correlation between LAI and each variable

**Fig. 6:**
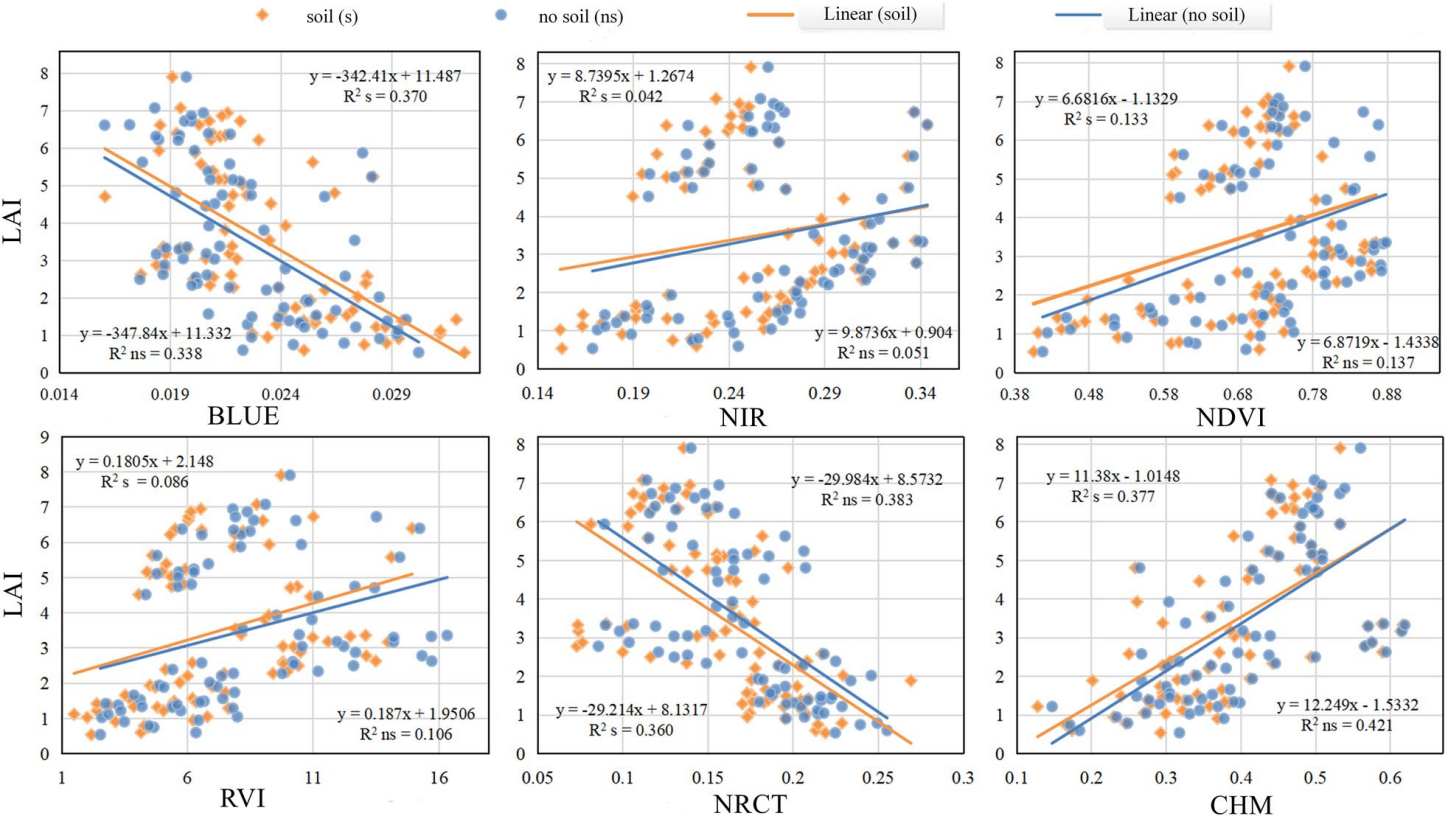
Correlation between LAI and various features with or without soil background. Ns and s denote the feature with no soil background and with soil background

Features were extracted from datasets with and without soil background to investigate the influence of soil background on LAI prediction under high-resolution UAV imagery. The correlation analysis experiments were carried out to obtain the R^2^ between each feature and LAI, and the results are shown in Fig. 6.

To begin, it can be seen that the correlation between LAI and various features differs with and without soil background, such as the correlation result between LAI and CHM (R^2^_ns_ = 0.421 and R^2^_s_ = 0.377). Second, the correlation between LAI and each feature is different, and the R^2^ between BLUE and CHM is higher relatively (BLUE: R^2^_ns_ = 0.338 and R^2^_s_ = 0.370, CHM: R^2^_ns_ = 0.421 and R^2^_s_ = 0.377). The correlation of LAI and VIs is poor (NDVI: R^2^_ns_ = 0.137 and R^2^_s_ = 0.133, RVI: R^2^_ns_ = 0.106 and R^2^_s_ = 0.086). It demonstrates that the correlation between LAI and each feature is not high whether there is soil background or not.

### LAI prediction model

RFR and SVR were employed for LAI prediction using canopy spectral, structure, and thermal features with or without soil background, respectively, the validation statistics of different models in Table 3.

**Table 3:** Validation statistics of different models for wheat LAI prediction

Feature	Metrics	Removing soil background		Retaining soil background	
		RFR	SVR	RFR	SVR
Sp	R^2^	0.746	0.679	0.684	0.539
	RMSE	1.185	1.233	1.441	1.472
Sp + Th	R^2^	0.689	0.701	0.573	0.536
	RMSE	1.391	1.176	1.604	1.474
Sp + St	R^2^	0.792	0.741	0.773	0.564
	RMSE	1.135	1.127	1.135	1.391
Sp + Th + St	R^2^	0.815	0.748	0.781	0.576
	RMSE	1.023	1.121	1.128	1.304

*Sp* spectral features, *St* structure features, *Th* thermal features

Firstly, the features extracted from the dataset without soil background are analyzed. It can be seen that when only spectral features are used, the range of R^2^ is 0.679–0.746 and the range of RMSE is 1.233–1.185. Compared with the blue or near-infrared band used alone in the subsection “Correlation between LAI and each variable”, R^2^ is increased by 0.25 or 0.62. It demonstrates that fusing all spectral features is superior to using any spectral feature alone, and the accuracy has been significantly improved.

**Fig. 7:**
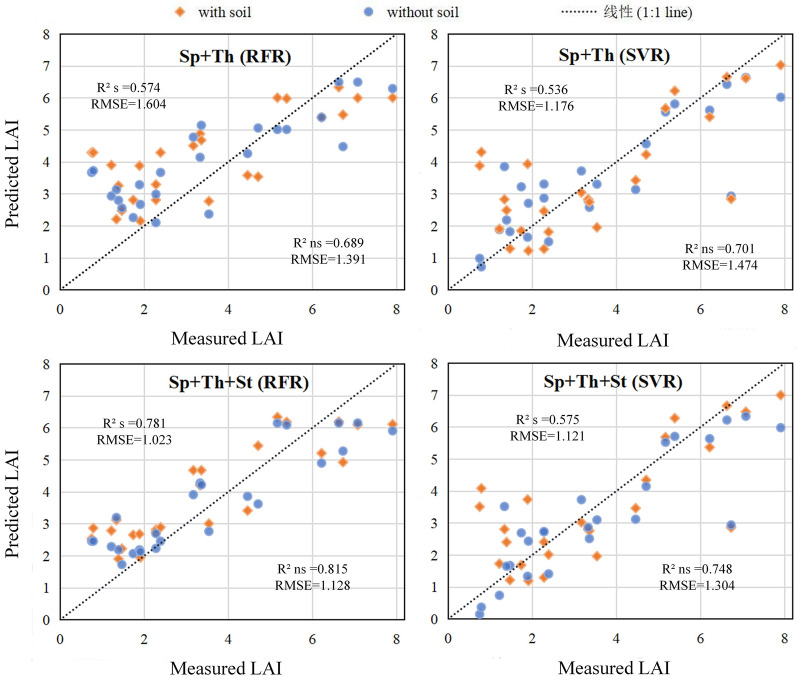
The validation scatter plots for measured versus prediction LAI

When compared to spectral features alone, combining spectral and thermal features slightly improve the accuracy of the SVR model (R^2^: 0.701 and RMSE: 1.176), R^2^ increases by 0.03, and RMSE decreases by 0.057. The prediction accuracy of the RFR model decreases (R^2^: 0.689 and RMSE: 1.391), R^2^ decreases by 0.11, and RMSE increases by 0.206. It is demonstrated that the combination of spectral and thermal features performs poorly in the LAI prediction model, as shown in Fig. 7.

When combining structural and spectral features, the prediction accuracy of both RFR and SVR models is improved compared with the spectral features alone and the combination of spectral and thermal features. Compared with spectral features alone, R^2^ of the RFR model increases by 0.05 and RMSE decreases by 0.05; R^2^ of the SVR model increases by 0.07, and RMSE decreases by 0.106. Compared with the combination of spectral and thermal features, R^2^ of the RFR model increases by 0.11 and RMSE decreases by 0.256, R^2^ of the SVR model increases by 0.04, and RMSE decreases by 0.049.

When all features were added to the model, the model's prediction accuracy improves to some extent, and the accuracy peaks (R^2^: 0.748–0.815 and RMSE: 1.023–1.121), as shown in Fig. 7. R^2^ increases by 0.069, and RMSE decreases by 0.162 compared to the RFR model using only spectral feature; R^2^ increases by 0.069, and RMSE decreases by 0.112 compared to the SVR model using only spectral feature. When combining spectral and thermal features, R^2^ increases by 0.126, and RMSE decreases by 0.368 for the RFR model; for the SVR model, R^2^ increases by 0.047, while RMSE decreases by 0.055. Compared to the combination of spectral and structural features, R^2^ increased by 0.023 and RMSE decreases by 0.112 for the RFR model, R^2^ increases by 0.007, and RMSE decreases by 0.006 for the SVR model. It can be seen that the model accuracy of the combination of all features is not improved compared to the model of the combination of spectral and structural features, indicating that adding thermal features to spectral and structural features has little effect on LAI prediction accuracy. Simultaneously, it is demonstrated that combining all features is superior to using only one or two features.

Similar to the model without soil background, the model's prediction accuracy with soil background is reduced by combining thermal and spectral features compared to the combination of spectral features. Combining spectral and structural features improves the model's accuracy using only spectral features (R^2^: 0.584–0.773 and RMSE: 1.135–1.391). Secondly, for the RFR model, the prediction accuracy of combining spectral, structural, and thermal features reaches the highest (R^2^ = 0.748, and RMSE = 1.128).

However, the model with soil background was different from the model without soil background. Whether RFR or SVR was used, the model without soil background was higher (R^2^: 0.679–0.746 and RMSE: 1.185–1.233). Similarly, for the combination of spectral and thermal features and the combination of spectral and structural features, the model without soil background has higher accuracy than the model with soil background (R^2^: 0.689–0.701, RMSE: 1.176–1.391, and R^2^: 0.741–0.746, RMSE: 1.135–1.127). When combining spectral, structure, and thermal features, compared with the model with soil background, the R^2^ of the RFR model without soil background increases from 0.781 to 0.815, and the RMSE decreases from 1.128 to 1.023; for the SVR model, R^2^ increases from 0.576 to 0.748 and RMSE decreases from 1.304 to 1.121. It is shown that the soil background can be removed well with the help of high-resolution UAV imagery. This operation retains the pure vegetation characteristics, avoids the interference of soil factors on the model, and improves the prediction accuracy of LAI.

### The importance of variables

**Fig. 8:**
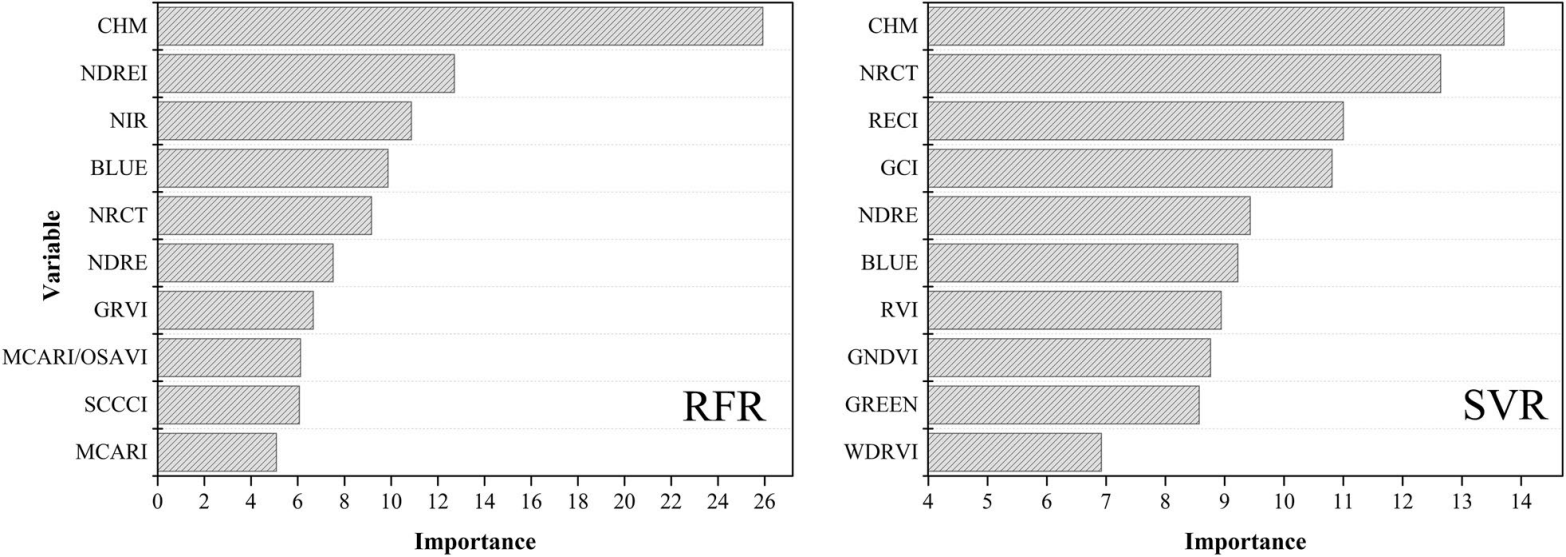
Top 10 features in importance

It can be seen that the highest prediction accuracy can be obtained based on the combination of spectral, structural, and thermal features without soil background. The importance of variables in RFR and SVR models was ranked based on the optimal feature combination, as shown in Fig. 8.

It could be seen that the score of CHM is the highest among the importance of variables in the RFR and SVR models, indicating that the structural feature plays an essential role in both models (Fig. 8). It is due to the structural feature being relatively independent of the other features in the model and contributing the most to the LAI estimation. In addition to the CHM, which has the highest importance score in the RFR model, NDREI, NIR, BLUE, and NRCT have higher importance scores. NRCT, RECI, GCI, and NDRE have higher importance scores except for CHM in the SVR model. The importance scores of thermal features and some VIs are high relatively, indicating that they performed well in LAI estimation.

It can be found that there are four common variables in the top ten variables of the two models, namely CHM, BLUE, NRCT, and NDRE. To quickly estimate the LAI of wheat, MLR was used based on these four common variables, and the multivariate linear formula was as follows.

5 $$y = - 390.65 * x_{BLUE} - 12.14 * x_{NDRE} - 3.2 * x_{NRCT} + \, 9.49 * x_{CHM} + 12.92$$

To better evaluate the effect of the MLR model in estimating LAI, RFR and SVR models are used simultaneously, the results are shown in Table 4.

**Table 4:** Verification accuracy of different regression methods

CHM + NRCT + BLUE + NDRE	MLR	RFR	SVR
R^2^	0.679	0.734	0.584
RMSE	1.231	1.156	1.413

**Fig. 9:**
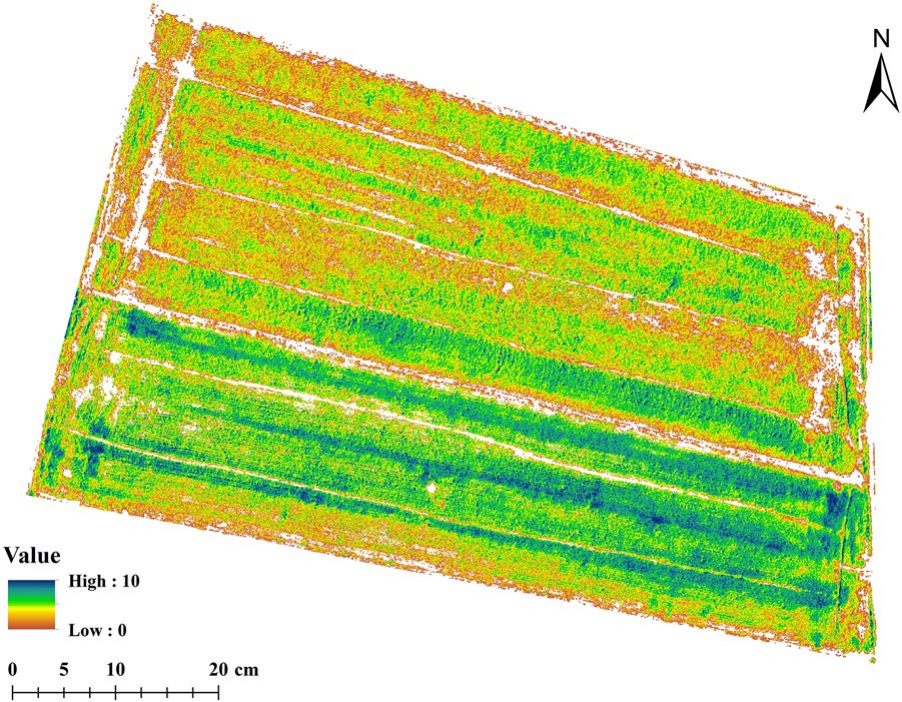
LAI prediction map derived when applying the MLR model to the 4 common variable images

It can be seen that the prediction accuracy of the SVR model based on four common variables is the lowest (R^2^ = 0.584 and RMSE = 1.413), and the prediction accuracy of the RFR model is the highest (R^2^ = 0.584 and RMSE = 1.156). High prediction accuracy can be obtained using the MLR model predicted LAI of four variables (R^2^ = 0.679 and RMSE = 1.231). In addition, compared with the prediction accuracy of the RFR model (R^2^ = 0.815 and RMSE = 1.023) and SVR model (R^2^ = 0.748 and RMSE = 1.121) using all the features without soil background, the prediction accuracy obtained by MLR is little different. Although the prediction accuracy of the MLR model is slightly lower than that of the RFR model, MLR is easier to operate, and the running time is shorter than RFR. It is demonstrated that to some extent, using four variables (CHM, BULE, NRCT, and NDRE), the MLR model can replace the RFR model using all features. It is also demonstrated that using fewer variables instead of all variables could achieve higher LAI prediction accuracy, reduce the system calculation time, and improve efficiency. At the same time, the MLR formula is used to predict LAI, and the prediction map is obtained, as shown in Fig. 9.

## Discussion

### Influence of soil background

Our study found that the soil background could reduce the accuracy of the LAI prediction., The accuracy improved significantly after removing the soil background, R^2^ raised by about 0.27, and RMSE fell by about 0.476. It could be because UAV imagery has the high spatial resolution, giving it an advantage over satellite images in distinguishing soil and vegetation. Previous research had also discovered the effect of soil background on estimating plant traits. Shu et al. [17] discovered that removing the soil background from 1.9 cm/pixel UAV hyperspectral imagery can effectively estimate the SPAD value of corn leaves, which is consistent with our findings. In recent years, some researchers have studied the effects of soil backgrounds on crop canopy reflectance spectrum and VIs. They found that VIs with no soil background can better reflect plant growth and ecological parameters [51, 52], indicating the importance and feasibility of removing the soil background of VIs, which is consistent with our findings.

Previous studies found that the influence of soil background with different brightness on canopy spectral information (such as NDVI) is different [53]. In terms of NDVI, the dark soil background reduced the overall reflectance of the canopy, while the bright soil background increased the reflectance of the canopy. As the brightness of the soil background increased from dark to bright, the value of NDVI showed a trend from high to low, allowing us to investigate further the influence of soil brightness on canopy spectral information. Furthermore, it is widely assumed that the influence of soil background on vegetation-soil systems has two components [11]. One factor is that soil components derived from mixed vegetation and soil pixels directly impact vegetation reflectance. The other factor is that multi-scattering between soil and vegetation indirectly affects the vegetation spectrum, which is more complex. We will further consider the impact of soil factors on vegetation spectral information in the future.

### Contribution of different features

Our study demonstrated that the fusion of multi-sensor synchronous observation data could achieve higher accuracy (R^2^ = 0.815 and RMSE = 1.023) than using only one feature, indicating the significance of feature fusion for wheat LAI prediction. Consistent with our findings, Maimaitijiang et al. [20] discovered that combining the soybean canopy's spectral, structural, thermal, and texture features, as opposed to using only one feature, can improve soybean yield prediction accuracy (R^2^ = 0.72 and RMSE = 478.9 kg/ha). Oliveira et al. [54] discovered that combining forage spectral and structural features could achieve higher prediction accuracy and accurate monitoring and prediction of forage yield. Combination of canopy spectral, structure, thermal and texture information contained in diverse sensor systems has the potential to improve plant trait estimations in a variety of agricultural applications over using features from a single sensor [55]. It may be attributed to the fact that is estimating plant traits, such as biomass or grain yield, from spectral information is hampered by asymptotic saturation observed from multi and hyperspectral optical sensors that do not account for three-dimensional (3D) structural information, especially among dense and heterogeneous canopies [56].

To begin, this study demonstrates that combining spectral and structural features can significantly improve prediction accuracy, which is consistent with previous studies that have shown the potential of coupling spectral and structural features in crop monitoring and grain yield [57–59]. One possible reason for this phenomenon is that the structural feature contains independent information about canopy growth and structure rather than those obtained from spectral features [25, 57]. Another possible reason is that the structure feature, to some extent, can overcome the problem of asymptotic saturation inherent in spectral features [55]. Secondly, the fusion of spectral, structural, and thermal features of canopy to estimate LAI had achieved better accuracy, which may be because the spectral, structural and thermal features of canopy provide unique and complementary information, which was consistent with previous studies [25]. It shows that the combination of canopy thermal, spectral, and structural features could improve the robustness of yield prediction under different weather conditions and crop development stages.

Previous research has shown that when all other factors are held constant, the greater the leaf area per unit surface area, or the greater the LAI value, the greater the water content of the crop itself [60, 61]. Because thermal features are related to leaf water content, pigment concentration, and canopy structure, LAI is closely associated with thermal features [62–64]. However, complex environmental conditions such as soil background, water availability, and atmospheric conditions all impact canopy temperature [65]. As a result, in this study, spectral and thermal features were used as input parameters of the model. Whether RFR or SVR was used, the accuracy will be reduced to some extent. This phenomenon could be caused by environmental factors such as average temperature, soil moisture, and organic matter composition in soil. In the follow-up research, it is necessary to further understand the relationship between canopy thermal feature and LAI, especially under various factors such as different plant species, development stages, environmental conditions, and interactions. It should be noted that adding thermal feature to the spectral feature reduced the accuracy. However, when ranking the importance of variables in the model after combining spectral, structural, and thermal features, it is clear that thermal feature had high significance. This phenomenon could be caused by the interaction of spectral, thermal, and structural features, emphasizing the importance of NRCT. Furthermore, NRCT was used in this study to quantify temperature. NRCT has been used to assess and forecast crop water status and traits. In general, NRCT requires the measured ground temperature to normalize. However, the canopy temperature was not calculated by the measured ground temperature due to external factors (environment and equipment, for example). Still, the NRCT was obtained directly by using statistical values in the thermal infrared image of the UAV. The measured ground temperature data will be used in the follow-up study to predict the LAI of wheat.

### Influence of high-resolution UAV image

Our study also proved that UAV remote sensing has the characteristics of high spatial resolution, so it has certain advantages in removing soil background. It may be because of the coarse spatial resolution and unsatisfactory time sampling of satellite imagery, which hinders its application in predicting plant traits. UAV remote sensing has been developing in recent years. Compared to airborne and satellite platforms, UAV remote sensing has the high spatial and spectral resolution and low cost. It has the high spatial resolution, which allows it to distinguish features visually. At the same time, its high spectral resolution improves the accuracy of feature spectral information, allowing for further research and analysis. Because of UAV remote sensing characteristics described above, UAV imagery could be widely used to estimate physical and chemical parameters of crops (such as LAI, N) and field-scale yield estimation, crop growth state monitoring, and other aspects [18, 55].

Since our study focused on investigating the impact of high spatial resolution combined with multi-sensor synchronous observation on LAI estimation, only UAV imagery with the high resolution of 1.49 cm/pixel is used for research. Previous research has shown that imagery with different spatial resolutions had different effects on the accuracy of plant trait estimation. For example, Guo et al. [66] used different flight altitudes to evaluate the impact of UAV imagery with different spatial resolutions on SPAD prediction. It was found that compared with imagery obtained with flight altitudes of 75 m (2.1 cm/pixel), 100 m (2.8 cm/pixel), and 125 m (3.4 cm/pixel), imagery with a flying height of 50 m (spatial resolution of 1.8 cm/pixel) could be used to estimate SPAD in leaves accurately. To further investigate the impact of UAV imagery with different spatial resolutions on LAI prediction, we resampled the imagery in this study into the 5 cm/pixel UAV imagery without soil background. After combining all the features, RFR was used to predict LAI. Compared with the R^2^ obtained from the imagery of 1.49 cm/pixel in this study, the R^2^ obtained from the 5 cm/pixel imagery modeling decreased by about 0.3–0.4, and the RMSE increased by about 2–3. It was demonstrated that using UAV imagery with higher spatial resolution can better help distinguish soil background and vegetation, which is beneficial in estimating wheat LAI.

### Uncertainty and outlook

Since the purpose of this study is to investigate the application potential of high spatial resolution of UAV imagery combined with multi-sensor synchronous observation in LAI estimation, RFR and SVR are used for all spectral, structural, and thermal features combinations. Of course, MLR is also used to estimate LAI, but due to the poor effect, it is not shown in this article; only the RFR and SVR methods with the best impact are shown. However, for a few essential features selected from all variables. The MLR method achieves satisfactory results, shortens the running time of the model, improves the efficiency, and provides an idea for rapid estimation of LAI of wheat.

In the past decade, the performance of deep learning developed from traditional neural networks has been significantly improved, surpassing the traditional models in the field of earth observation [67]. However, it has certain limitations, for example, a large number of training data are needed to effectively converge the deep learning model to obtain the optimal model parameters. Shallow machine learning regression models, which include RFR, SVR, ANN, do not need a lot of training data. Compared with traditional algorithms, the machine learning regression model can effectively use data when dealing with high-dimensional and complex data to obtain higher prediction accuracy of the model [18, 68]. Because our study focused on the influencing factors of wheat LAI prediction (such as soil), we only used commonly used machine learning methods for data fusion (RFR, SVR, and MLR). At the same time, our study demonstrated that LAI of wheat could be effectively predicted under the data fusion framework of UAV high-resolution imagery and multi-sensor synchronous observation. Previous research was consistent with our findings, indicating that plant traits could be effectively predicted under this framework. For example, Oliveira et al. [54] extracted the height and spectral information of forage from UAV visible and hyperspectral imagery respectively and fused the features by RFR and MLR methods. Han et al. [69] used the structural and spectral information provided by UAV imagery, combined with RFR, SVR and MLR to estimate corn biomass. In addition, this study found that no matter which features fusion is used, RFR performs best in LAI prediction, consistent with previous studies on other traits of crops [18, 70]. Zha et al. [70] found RF performed better than SVR, MLR, and ANN on predicting nitrogen content in rice using spectral features. Previous studies have also shown that SVR is a new generation of machine learning algorithms based on statistical learning theory. The advantages of SVR are mainly reflected in solving linear inseparable problems. It solves the inner product operation in high-dimensional space by introducing kernel function to solve the nonlinear problem [48, 49]. The kernel functions of SVR include radial basis function, polynomial, sigmoid kernel, and linear kernel function. Because of many experiments and the advantages of fewer parameters and fast speed, the linear kernel function with the best performance was selected to predict the LAI of wheat.

Previous research had shown that UAV imagery from the entire crop growth cycle could help us determine which period can accurately predict crop traits and growth in the early stages [19]. However, this study only used the imagery of wheat at the heading stage due to the weather and crew availability. In future research, we will consider the prediction of LAI throughout the wheat growth cycle.

## Conclusions

Under the data fusion framework of UAV high-resolution imagery and multi-sensor synchronous observation, the influence of soil background on LAI estimation was investigated. The following conclusions could be drawn: (1) the soil background will affect the estimation of wheat LAI. The high-resolution UAV imagery can effectively remove the soil background, and the prediction accuracy is significantly improved after removal, R^2^ is increased by about 30.78%, and RMSE is decreased by about 20.22%. (2) In addition to the commonly used structural feature (CHM), the temperature feature (NRCT) is also important for the prediction of LAI of wheat. (3) A simple method of combining multiple features for wheat LAI prediction, that is, using machine learning to screen out a few variables and then using one method, high prediction accuracy could be achieved (R^2^ = 0.679 and RMSE = 1.231).

The finding indicates that the method based on UAV high-resolution imagery combined with multi-sensor data fusion has a great potential for crop LAI estimation. Furthermore, it has guiding significance for high-precision LAI rapid prediction using UAV remote sensing technology and a reference value for precision agriculture promotion and application. However, to further assess its robustness, this method will need to be tested on different crop types at different stages of development and under different environmental conditions in future research.

## Data Availability

The remotely sensed and field sampling data used in this study is available from the corresponding author on reasonable request.

## References

[1] Qiu B, Luo Y, Tang Z (2017). Winter wheat mapping combining variations before and after estimated heading dates. ISPRS J Photogramm.

[2] Nasrallah A, Baghdadi N, El Hajj M (2019). Sentinel-1 Data for winter wheat phenology monitoring and mapping. Remote Sens.

[3] Zhong L, Hu L, Zhou H, Tao X (2019). Deep learning based winter wheat mapping using statistical data as ground references in kansas and northern texas,US. Remote Sens Environ.

[4] Liu X, Jin J, Herbert SJ, Zhang Q, Wang G (2005). Yield components, dry matter, LAI and LAD of soybeans in Northeast China. Field Crop Res.

[5] Zhou X, Zheng HB, Xu XQ (2017). Predicting grain yield in rice using multi-temporal vegetation indices from UAV-based multispectral and digital imagery. ISPRS J Photogramm.

[6] Qiao K, Zhu W, Xie Z, Li P (2019). estimating the seasonal dynamics of the leaf area index using piecewise LAI-VI relationships based on phenophases. Remote Sens.

[7] Goude M, Nilsson U, Holmström E (2019). Comparing direct and indirect leaf area measurements for scots pine and norway spruce plantations in sweden. Eur J Forest Res.

[8] Yan G, Hu R, Luo J (2019). Review of indirect optical measurements of leaf area index: recent advances, challenges, and perspectives. Agr Forest Meteorol.

[9] Weissa M, Jacobb F, Duveillerc G (2020). Remote sensing for agricultural applications: a meta-review. Remote Sens Environ.

[10] WATSON DJ (1947). Comparative physiological studies on the growth of field crops. Ann Bot.

[11] Yu K, Lenz-Wiedemann V, Chen X, Bareth G (2014). Estimating leaf chlorophyll of barley at different growth stages using spectral indices to reduce soil background and canopy structure effects. ISPRS J Photogramm.

[12] Herrmann I, Pimstein A, Karnieli A (2011). LAI assessment of wheat and potato crops by VENμS and Sentinel-2 bands. Remote Sens Environ.

[13] Kamenova I, Dimitrov P (2021). Evaluation of sentinel-2 vegetation indices for prediction of LAI, fAPAR and fcover of winter wheat in bulgaria. Eur J Remote Sens.

[14] Meyer LH, Heurich M, Beudert B, Premier J, Pflugmacher D (2019). Comparison of landsat-8 and sentinel-2 data for estimation of leaf area index in temperate forests. Remote Sens.

[15] Mathews A, Jensen J (2013). Visualizing and quantifying vineyard canopy lai using an unmanned aerial vehicle (UAV) collected high density structure from motion point cloud. Remote Sens.

[16] Zhang S, Zhao G, Lang K (2019). Integrated satellite, unmanned aerial vehicle (UAV) and ground inversion of the spad of winter wheat in the reviving stage. Sensors.

[17] Shu M, Zuo J, Shen M (2021). Improving the estimation accuracy of SPAD values for maize leaves by removing UAV hyperspectral image backgrounds. Int J Remote Sens.

[18] Lee H, Wang J, Leblon B (2020). Using linear regression, random forests, and support vector machine with unmanned aerial vehicle multispectral images to predict canopy nitrogen weight in corn. Remote Sens.

[19] Yang K, Gong Y, Fang S (2021). Combining spectral and texture features of UAV images for the remote estimation of rice lai throughout the entire growing season. Remote Sens.

[20] Maimaitijiang M, Sagan V, Sidike P (2020). Soybean yield prediction from UAV using multimodal data fusion and deep learning. Remote Sens Environ.

[21] Schirrmann M, Giebel A, Gleiniger F (2016). Monitoring agronomic parameters of winter wheat crops with low-cost UAV imagery. Remote Sens.

[22] Hunt ER, Cavigelli M, Daughtry CST, Mcmurtrey JE, Walthall CL (2005). Evaluation of digital photography from model aircraft for remote sensing of crop biomass and nitrogen status. Precis Agric.

[23] Primicerio J, Di Gennaro SF, Fiorillo E (2012). A flexible unmanned aerial vehicle for precision agriculture. Precis Agric.

[24] Simpson JE, Holman F, Nieto H (2021). High spatial and temporal resolution energy flux mapping of different land covers using an off-the-shelf unmanned aerial system. Remote Sens.

[25] Rischbeck P, Elsayed S, Mistele B (2016). Data fusion of spectral, thermal and canopy height parameters for improved yield prediction of drought stressed spring barley. Eur J Agron.

[26] Fieuzal R, Marais Sicre C, Baup F (2017). Estimation of corn yield using multi-temporal optical and radar satellite data and artificial neural networks. Int J Appl Earth Obs.

[27] Aghighi H, Azadbakht M, Ashourloo D, Shahrabi HS, Radiom S (2018). Machine learning regression techniques for the silage maize yield prediction using time-series images of landsat 8 OLI. IEEE J Stars.

[28] Swatantran A, Dubayah R, Goetz S (2012). Mapping migratory bird prevalence using remote sensing data fusion. PLoS ONE.

[29] Maimaitijiang M, Sagan V, Sidike P (2020). Crop monitoring using satellite/UAV data fusion and machine learning. Remote Sens.

[30] Jin Z, Azzari G, Lobell DB (2017). Improving the accuracy of satellite-based high-resolution yield estimation: a test of multiple scalable approaches. Agr Forest Meteorol.

[31] Tucker CJ (1979). Red and photographic infrared linear combinations for monitoring vegetation. Remote Sens Environ.

[32] Gitelson AA (2005). Remote estimation of canopy chlorophyll content in crops. Geophys Res Lett.

[33] Rouse JR, Haas R, Schell J, Deering D (1974). Monitoring Vegetation Systems in the Grreat Plains with ERTS.

[34] Gitelson AA, Gritz Y, Merzlyak MN (2003). Relationships between leaf chlorophyll content and spectral reflectance and algorithms for non-destructive chlorophyll assessment in higher plant leaves. J Plant Physiol.

[35] Gitelson AA, Merzlyak MN (1997). Remote estimation of chlorophyll content in higher plant leaves. Int J Remote Sens.

[36] Hassan M, Yang M, Rasheed A (2018). Time-series multispectral indices from unmanned aerial vehicle imagery reveal senescence rate in bread wheat. Remote Sens.

[37] Raper TB, Varco JJ (2015). Canopy-scale wavelength and vegetative index sensitivities to cotton growth parameters and nitrogen status. Precis Agric.

[38] Rondeaux G, Steven M, Baret F (1996). Optimization of soil-adjusted vegetation indices. Remote Sens Environ.

[39] Daughtry CST, Walthall CL, Kim MS, de Colstoun EB, McMurtrey JE (2000). Estimating corn leaf chlorophyll concentration from leaf and canopy reflectance. Remote Sens Environ.

[40] Haboudane D, Miller JR, Tremblay N, Zarco-Tejada PJ, Dextraze L (2002). Integrated narrow-band vegetation indices for prediction of crop chlorophyll content for application to precision agriculture. Remote Sens Environ.

[41] Gitelson AA (2004). Wide dynamic range vegetation index for remote quantification of biophysical characteristics of vegetation. J Plant Physiol.

[42] Elsayed S, Rischbeck P, Schmidhalter U (2015). Comparing the performance of active and passive reflectance sensors to assess the normalized relative canopy temperature and grain yield of drought-stressed barley cultivars. Field Crop Res.

[43] Bending J, Bolten A, Bsreth G (2013). UAV-based imaging for multi-temporal, very high resolution crop surface models to monitor crop growth variability. Photogramm Fernerkun.

[44] Zhao K, Suarez JC, Garcia M (2018). Utility of multitemporal lidar for forest and carbon monitoring: Tree growth, biomass dynamics, and carbon flux. RemotE Sens Environ.

[45] Elsayed S, Elhoweity M, Ibrahim HH (2017). Thermal imaging and passive reflectance sensing to estimate the water status and grain yield of wheat under different irrigation regimes. Agr Water Manage.

[46] Breiman L (2001). Random Forests. Mach Learn.

[47] Belgiu M, Drăguţ L (2016). Random forest in remote sensing: A review of applications and future directions. ISPRS J Photogramm.

[48] Che J, Wang J (2014). Short-term load forecasting using a kernel-based support vector regression combination model. Appl Energ.

[49] Jiang H, Rusuli Y, Amuti T, He Q (2019). Quantitative assessment of soil salinity using multi-source remote sensing data based on the support vector machine and artificial neural network. Int J Remote Sens.

[50] Abrougui K, Gabsi K, Mercatoris B (2019). Prediction of organic potato yield using tillage systems and soil properties by artificial neural network (ANN) and multiple linear regressions (MLR). Soil Tillage Res.

[51] Gao X, Huete AR, Ni W, Miura T (2000). optical-biophysical relationships of vegetation spectra without background contamination. Remote Sens Environ.

[52] Huete AR, Tucker CJ (1991). Investigation of soil influences in AVHRR red and near-infrared vegetation index imagery. Int J Remote Sens.

[53] Bach H, Verhoef W (2003). Sensitivity studies on the effect of surface soil moisture on canopy reflectance using the radiative transfer model GeoSAIL. IEEE.

[54] Oliveira RA, Näsi R, Niemeläinen O (2020). Machine learning estimators for the quantity and quality of grass swards used for silage production using drone-based imaging spectrometry and photogrammetry. Remote Sens Environ.

[55] Maimaitijiang M, Ghulam A, Sidike P (2017). Unmanned aerial system (UAS)-based phenotyping of soybean using multi-sensor data fusion and extreme learning machine. ISPRS J Photogramm.

[56] Greaves HE, Vierling LA, Eitel JUH (2015). Estimating aboveground biomass and leaf area of low-stature Arctic shrubs with terrestrial LiDAR. Remote Sens Environ.

[57] Stanton C, Starek MJ, Elliott N (2017). Unmanned aircraft system-derived crop height and normalized difference vegetation index metrics for sorghum yield and aphid stress assessment. J Appl Remote Sens.

[58] Bendig J, Yu K, Aasen H (2015). Combining UAV-based plant height from crop surface models, visible, and near infrared vegetation indices for biomass monitoring in barley. Int J Appl Earth Obs.

[59] Geipel J, Link J, Claupein W (2014). Combined spectral and spatial modeling of corn yield based on aerial images and crop surface models acquired with an unmanned aircraft system. Remote Sens.

[60] Hansen PM, Schjoerring JK (2003). Reflectance measurement of canopy biomass and nitrogen status in wheat crops using normalized difference vegetation indices and partial least squares regression. Remote Sens Environ.

[61] de Jong SM, Addink EA, Doelman JC (2014). Detecting leaf-water content in Mediterranean trees using high-resolution spectrometry. Int J Appl Earth Obs.

[62] Elarab M, Ticlavilca AM, Torres-Rua AF, Maslova I, McKee M (2015). Estimating chlorophyll with thermal and broadband multispectral high resolution imagery from an unmanned aerial system using relevance vector machines for precision agriculture. Int J Appl Earth Obs.

[63] Neinavaz E, Skidmore AK, Darvishzadeh R, Groen TA (2016). Retrieval of leaf area index in different plant species using thermal hyperspectral data. ISPRS J Photogramm.

[64] Abu-Hamdeh NH (2003). Thermal properties of soils as affected by density and water content. Biosyst Eng.

[65] Aubrecht DM, Helliker BR, Goulden ML (2016). Continuous, long-term, high-frequency thermal imaging of vegetation: Uncertainties and recommended best practices. Agr Forest Meteorol.

[66] Guo Y, Yin G, Sun H (2020). Scaling effects on chlorophyll content estimations with rgb camera mounted on a uav platform using machine-learning methods. Sensors.

[67] Kamilaris A, Prenafeta-Boldú FX (2018). Deep learning in agriculture: a survey. Comput Electron Agr.

[68] Holloway J, Mengersen K (2018). Statistical machine learning methods and remote sensing for sustainable development goals: a review. Remote Sens.

[69] Han L, Yang G, Dai H (2019). Modeling maize above-ground biomass based on machine learning approaches using UAV remote-sensing data. Plant Methods.

[70] Zha H, Miao Y, Wang T (2020). Improving unmanned aerial vehicle remote sensing-based rice nitrogen nutrition index prediction with machine learning. Remote Sens.

